# Supplementary material for: Genomics of cellular proliferation in periodic environmental fluctuations
Source: Mol Syst Biol. 2018 Mar 5;14(3):e7823. doi: 10.15252/msb.20177823 (PMC5836541; doi:10.15252/msb.20177823)
Supplement: Supplementary file 1 — Appendix [file MSB-14-e7823-s001.pdf]

APPENDIX

Genomics of cellular proliferation in periodic environmental fluctuations

Jérôme Salignon*, Magali Richard*, Etienne Fulcrand, Hélène Duplus-Bottin and Gaël Yvert#

Laboratory of Biology and Modeling of the Cell, Ecole Normale Supérieure de Lyon, CNRS, Université Claude Bernard de Lyon, Université de Lyon, 69007 Lyon; France.

*) these authors contributed equally to this work

#) corresponding author, Gael.Yvert@ens-lyon.fr

TABLE OF CONTENTS

Appendix Figure S1... p2
Appendix Figure S2... p3-13
Appendix Figure S3... p14-15
Appendix Figure S4... p16-17
Appendix Figure S5... p18

Appendix Table S1.. p19
Appendix Table S2.. p20-21
Appendix Table S3.. p22
Appendix Table S4.. p22
Appendix Table S5.. p22
Appendix Table S6.. p23
Appendix Table S7.. p23

References.. p24

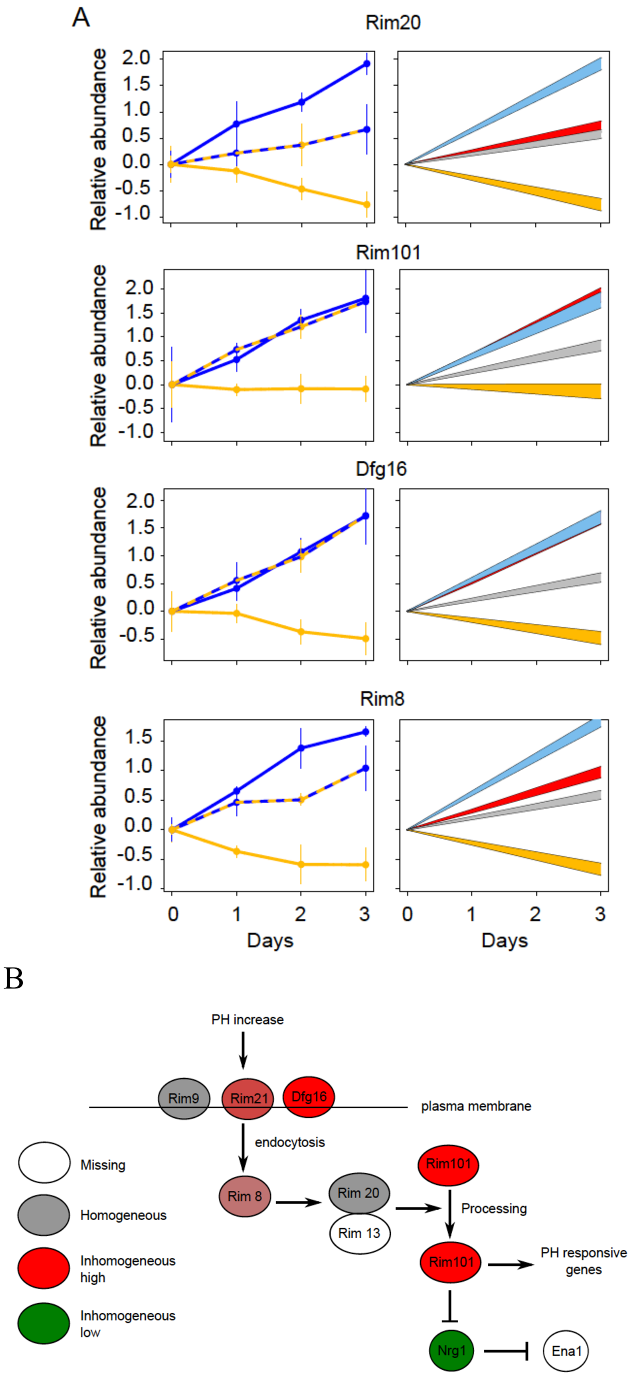

Appendix Figure S1. BAR-seq fitness profile of mutants of the Rim101 pathway. (**A**) For mutants of the Rim101 pathway available in our data is shown their time-course abundance (left) and their fitted Generalized linear models (right), as in Figure 1. (**B**) Schematic representation of the pathway with colors corresponding to the level of fitness inhomogeneity of each member.

Appendix Figure S2. Time-course of mutant abundance, for mutants analyzed by BAR-Seq and individual competition assays.

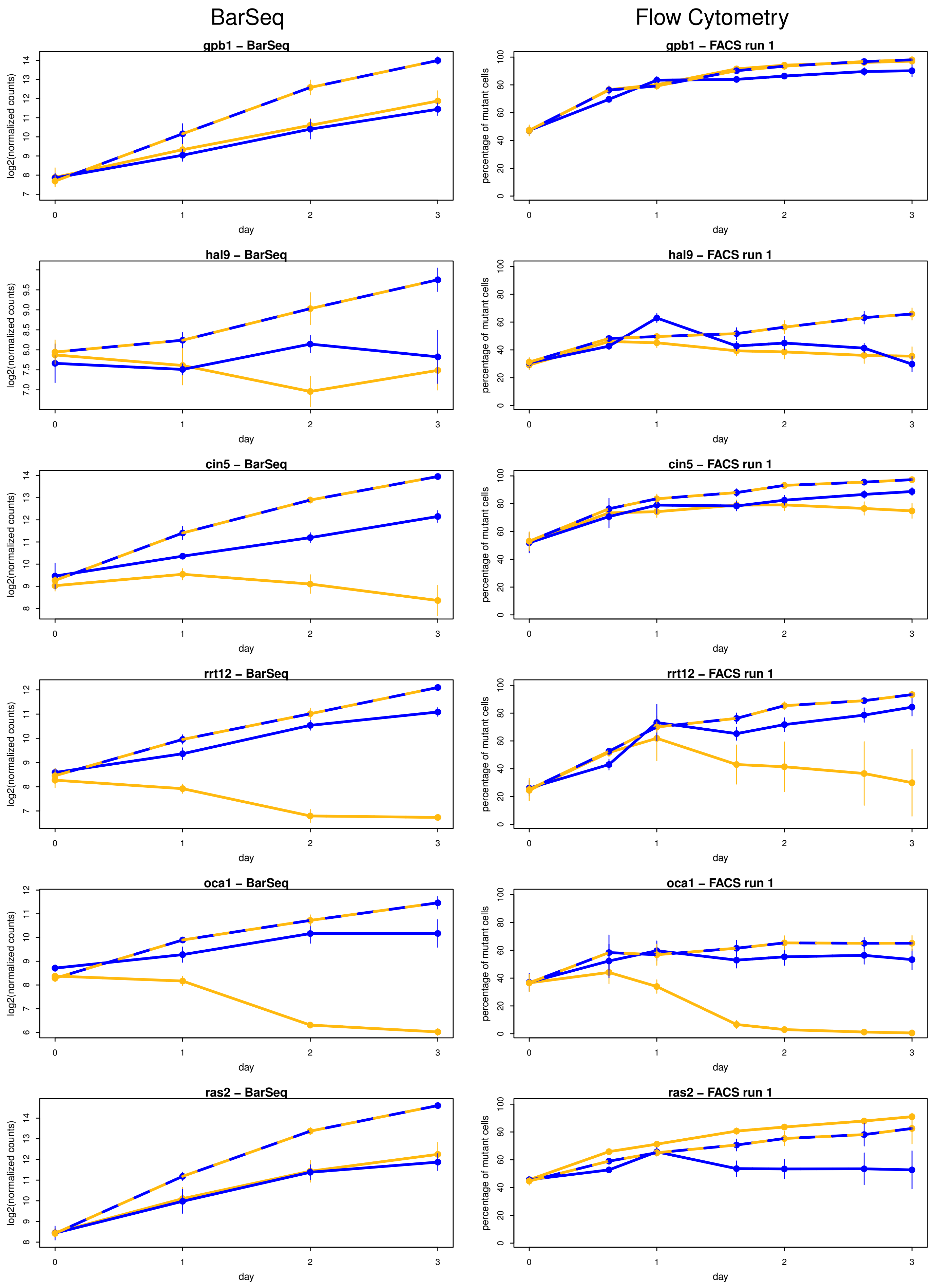

BarSeq
Flow Cytometry
gpb1 – BarSeq
gpb1 – FACS run 1
hal9 – BarSeq
hal9 – FACS run 1
cin5 – BarSeq
cin5 – FACS run 1
rrt12 – BarSeq
rrt12 – FACS run 1
oca1 – BarSeq
oca1 – FACS run 1
ras2 – BarSeq
ras2 – FACS run 1
log2(normalized counts)
percentage of mutant cells
day

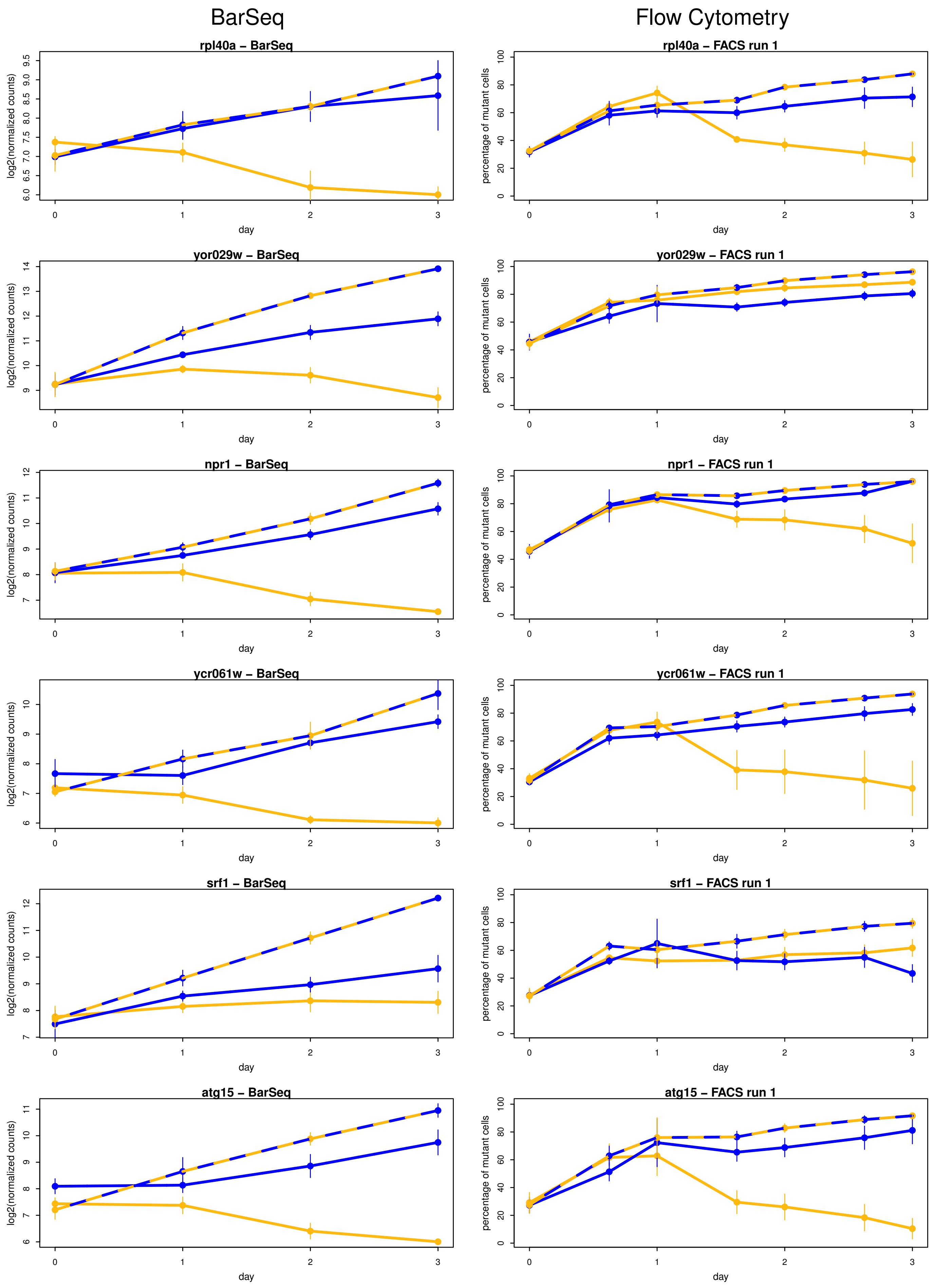
BarSeq
Flow Cytometry
rpl40a – BarSeq
rpl40a – FACS run 1
yor029w – BarSeq
yor029w – FACS run 1
npr1 – BarSeq
npr1 – FACS run 1
ycr061w – BarSeq
ycr061w – FACS run 1
srf1 – BarSeq
srf1 – FACS run 1
atg15 – BarSeq
atg15 – FACS run 1
log2(normalized counts)
percentage of mutant cells
day

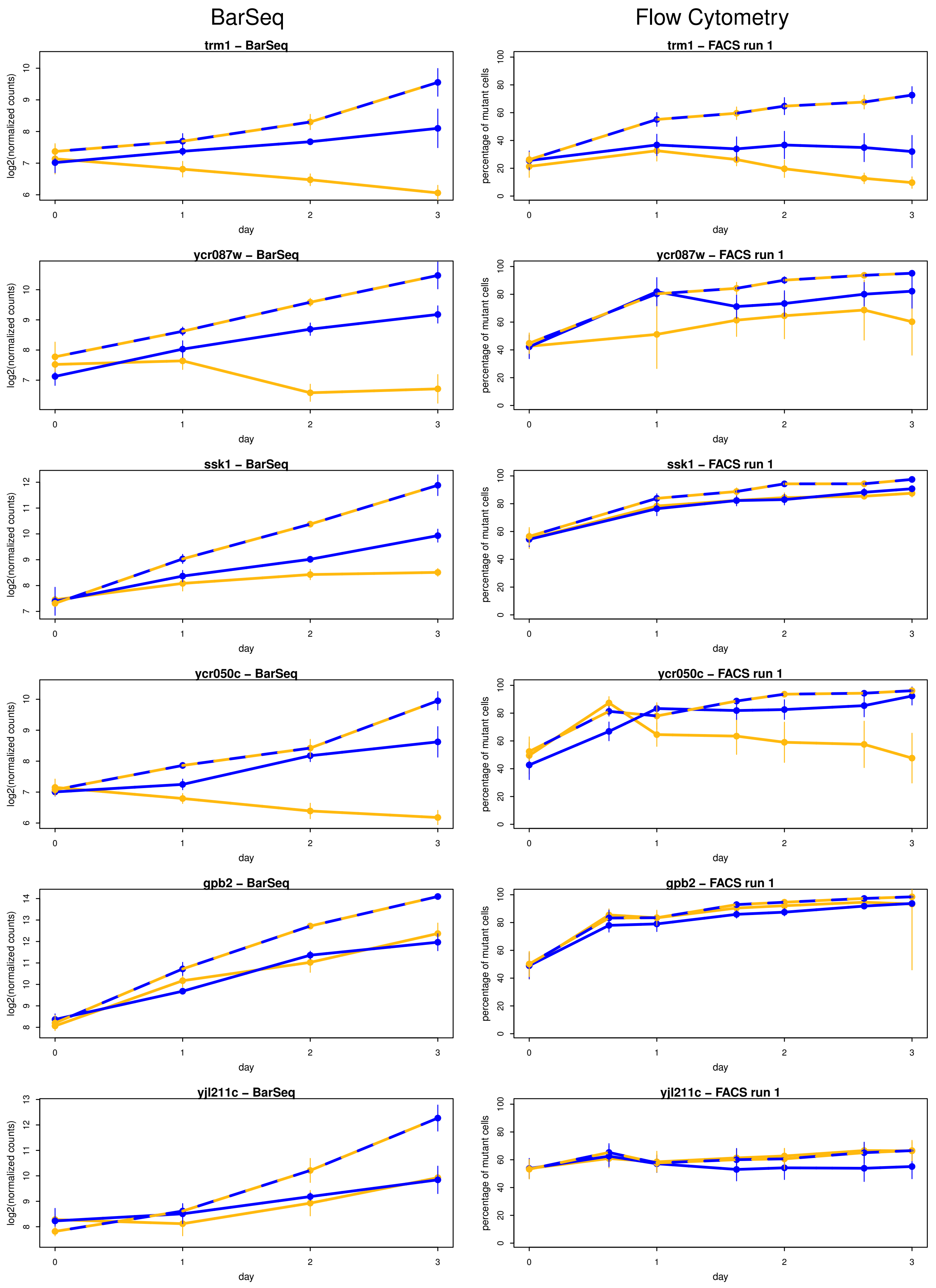
BarSeq
Flow Cytometry
trm1 – BarSeq
trm1 – FACS run 1
ycr087w – BarSeq
ycr087w – FACS run 1
ssk1 – BarSeq
ssk1 – FACS run 1
ycr050c – BarSeq
ycr050c – FACS run 1
gpb2 – BarSeq
gpb2 – FACS run 1
yjl211c – BarSeq
yjl211c – FACS run 1
log2(normalized counts)
percentage of mutant cells
day

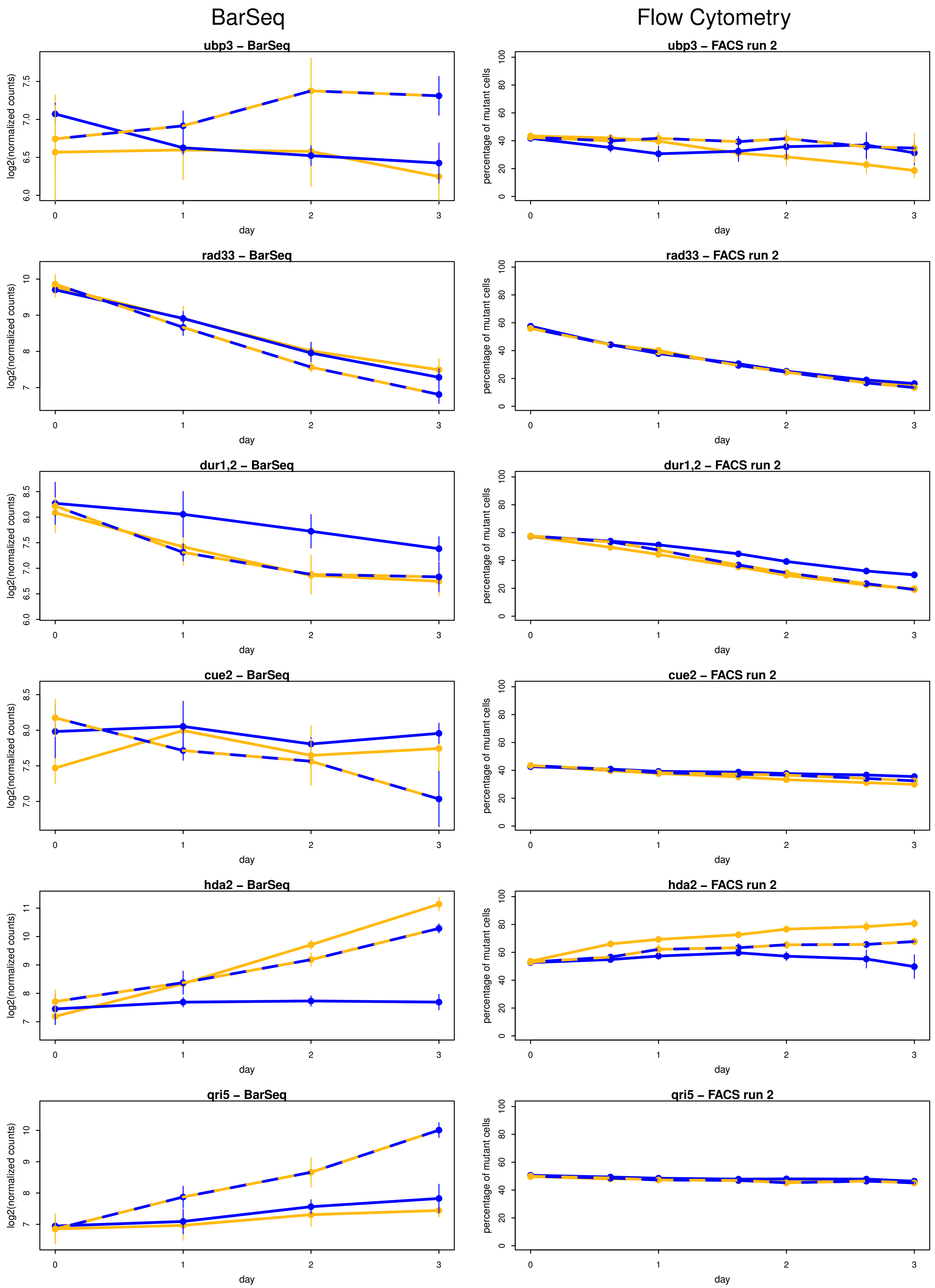
BarSeq
Flow Cytometry
ubp3 – BarSeq
ubp3 – FACS run 2
rad33 – BarSeq
rad33 – FACS run 2
dur1,2 – BarSeq
dur1,2 – FACS run 2
cue2 – BarSeq
cue2 – FACS run 2
hda2 – BarSeq
hda2 – FACS run 2
gri5 – BarSeq
gri5 – FACS run 2
log2(normalized counts)
percentage of mutant cells
day

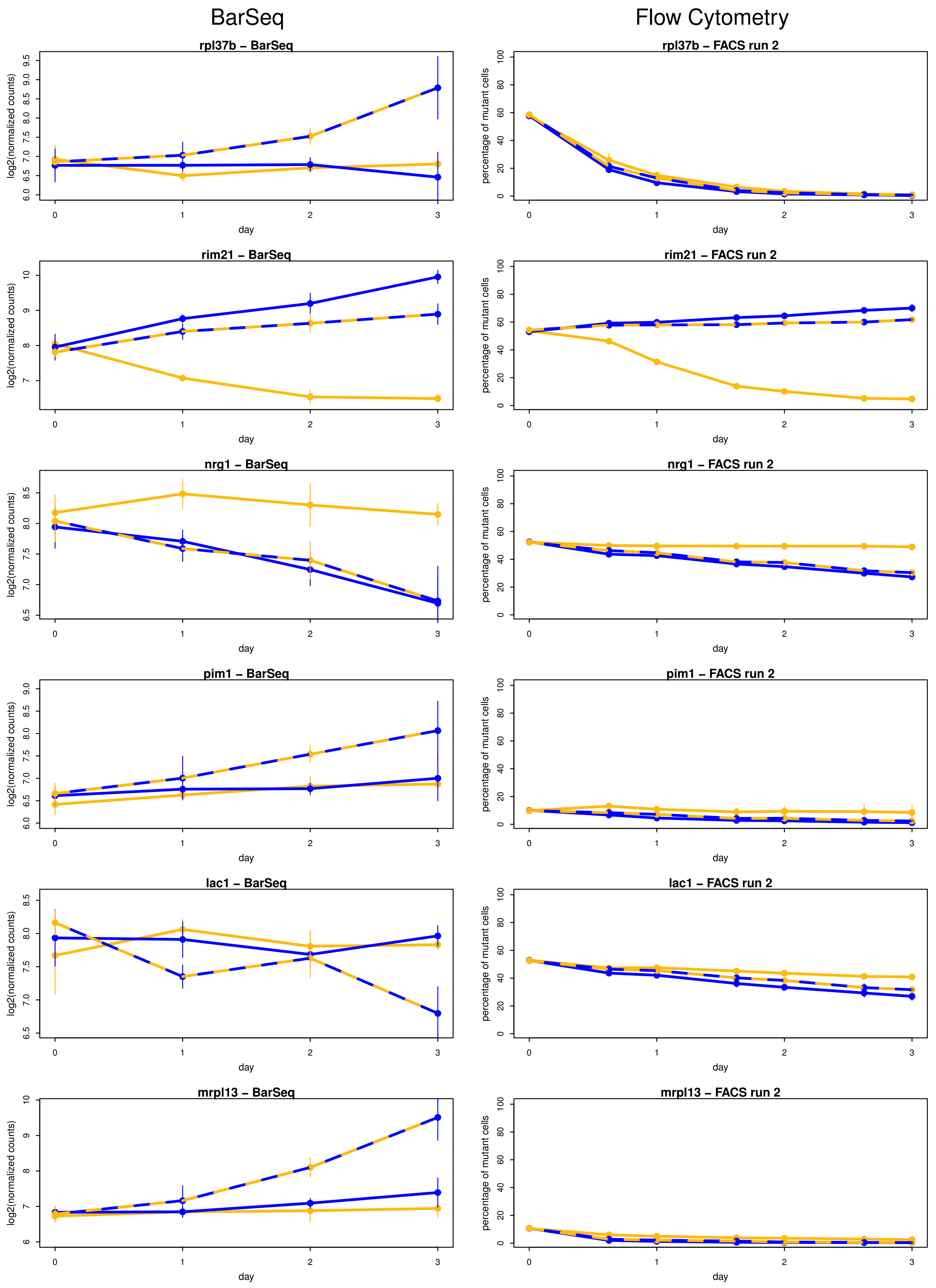

BarSeq
Flow Cytometry
rpl37b – BarSeq
rpl37b – FACS run 2
rim21 – BarSeq
rim21 – FACS run 2
nrg1 – BarSeq
nrg1 – FACS run 2
pim1 – BarSeq
pim1 – FACS run 2
lac1 – BarSeq
lac1 – FACS run 2
mrpl13 – BarSeq
mrpl13 – FACS run 2
log2(normalized counts)
percentage of mutant cells
day

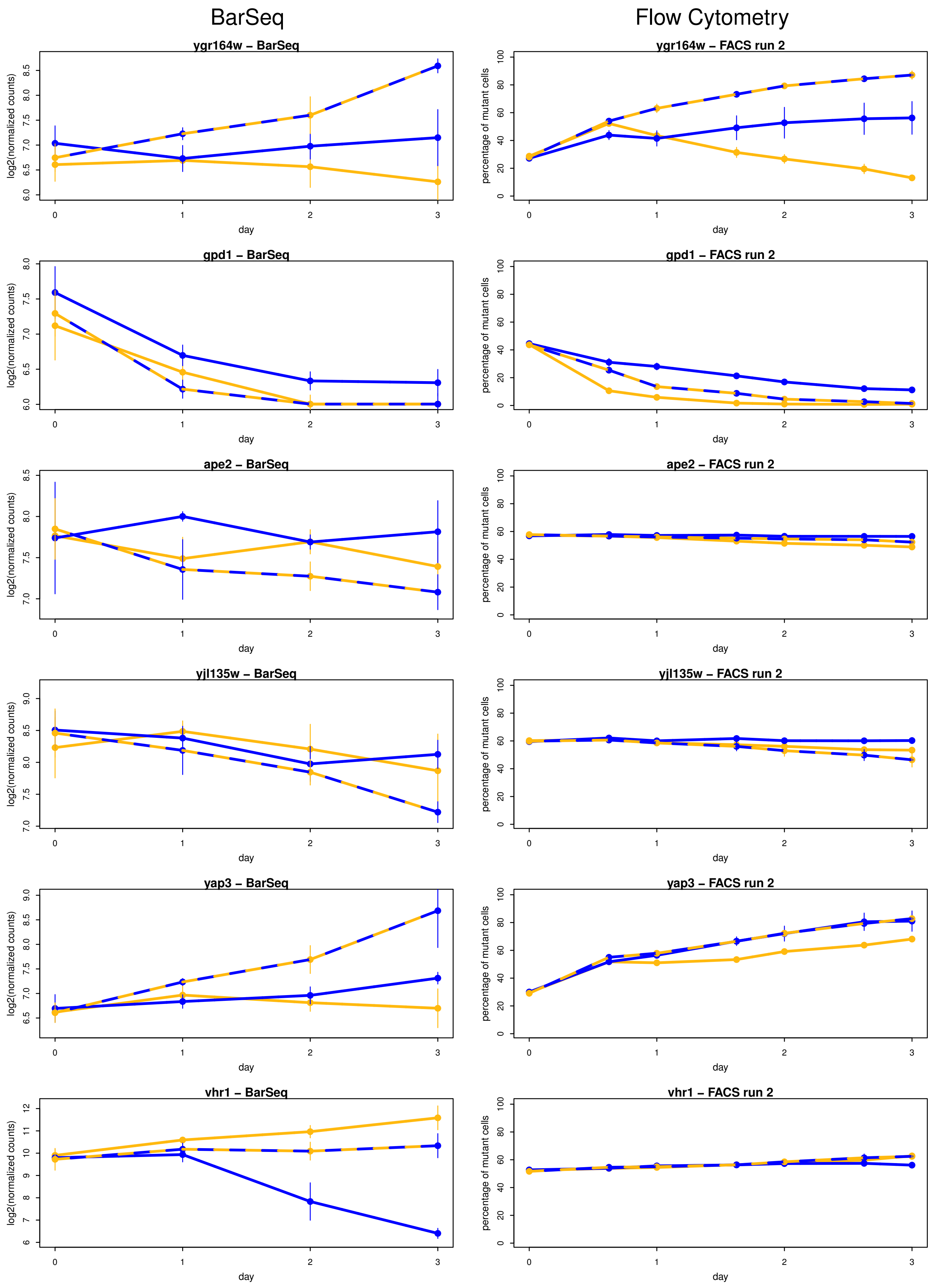
BarSeq
Flow Cytometry
ygr164w – BarSeq
ygr164w – FACS run 2
gpd1 – BarSeq
gpd1 – FACS run 2
ape2 – BarSeq
ape2 – FACS run 2
yjl135w – BarSeq
yjl135w – FACS run 2
yap3 – BarSeq
yap3 – FACS run 2
vhr1 – BarSeq
vhr1 – FACS run 2
log2(normalized counts)
percentage of mutant cells
day

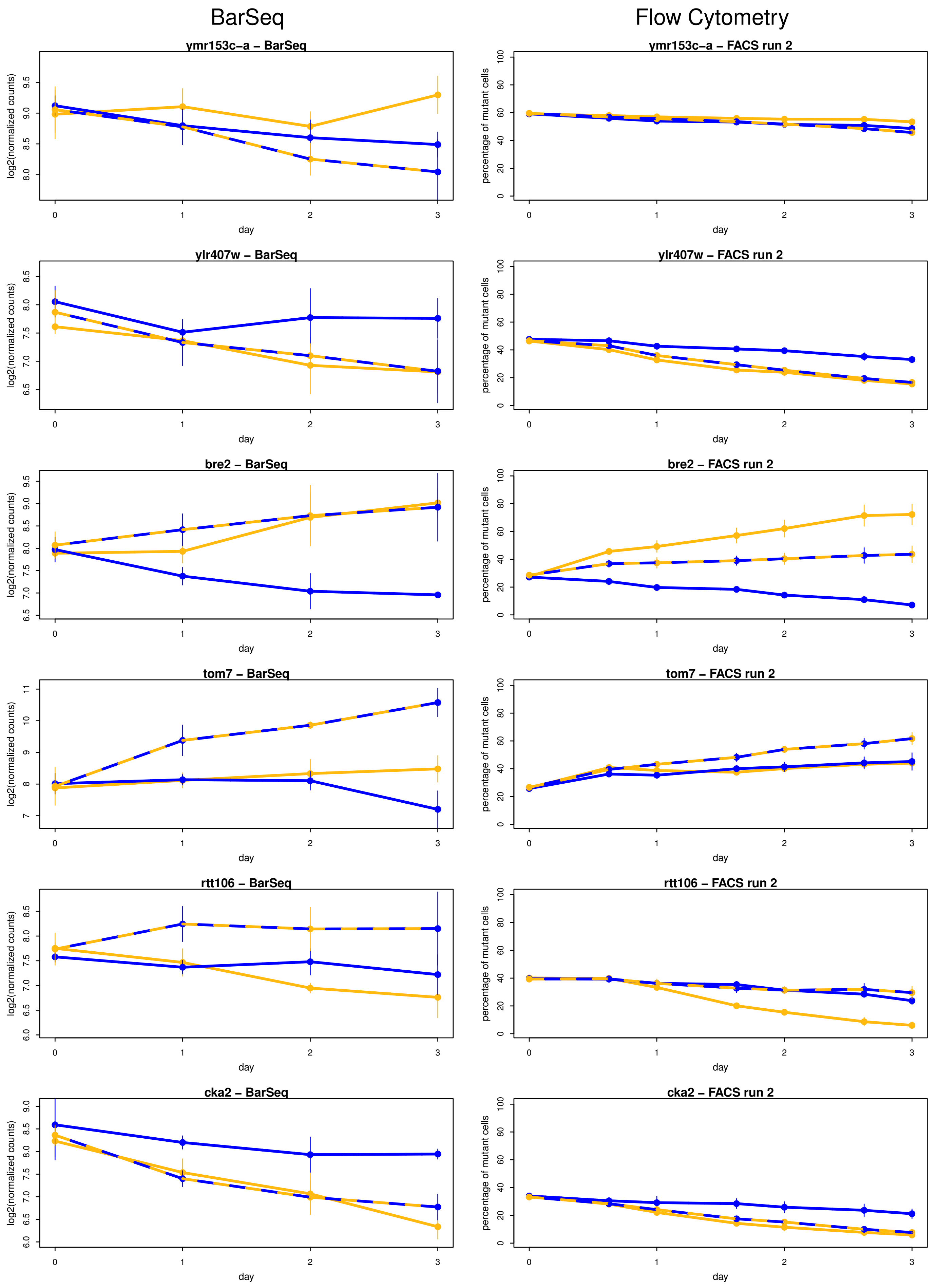
BarSeq
Flow Cytometry
ymr153c–a – BarSeq
ymr153c–a – FACS run 2
ylr407w – BarSeq
ylr407w – FACS run 2
bre2 – BarSeq
bre2 – FACS run 2
tom7 – BarSeq
tom7 – FACS run 2
rtt106 – BarSeq
rtt106 – FACS run 2
cka2 – BarSeq
cka2 – FACS run 2
log2(normalized counts)
percentage of mutant cells
day

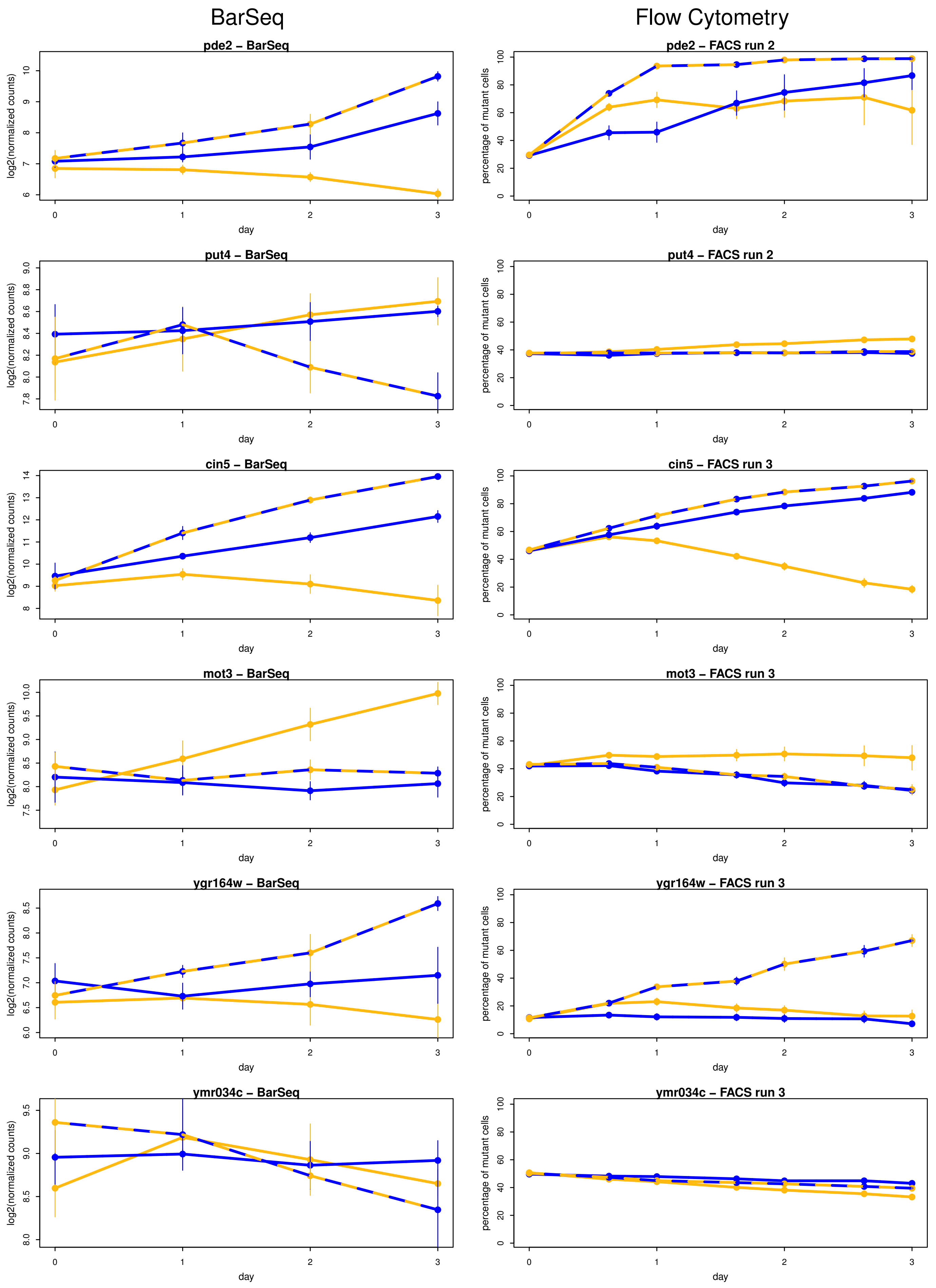
BarSeq
Flow Cytometry
pde2 – BarSeq
pde2 – FACS run 2
put4 – BarSeq
put4 – FACS run 2
cin5 – BarSeq
cin5 – FACS run 3
mot3 – BarSeq
mot3 – FACS run 3
ygr164w – BarSeq
ygr164w – FACS run 3
ymr034c – BarSeq
ymr034c – FACS run 3
log2(normalized counts)
percentage of mutant cells
day

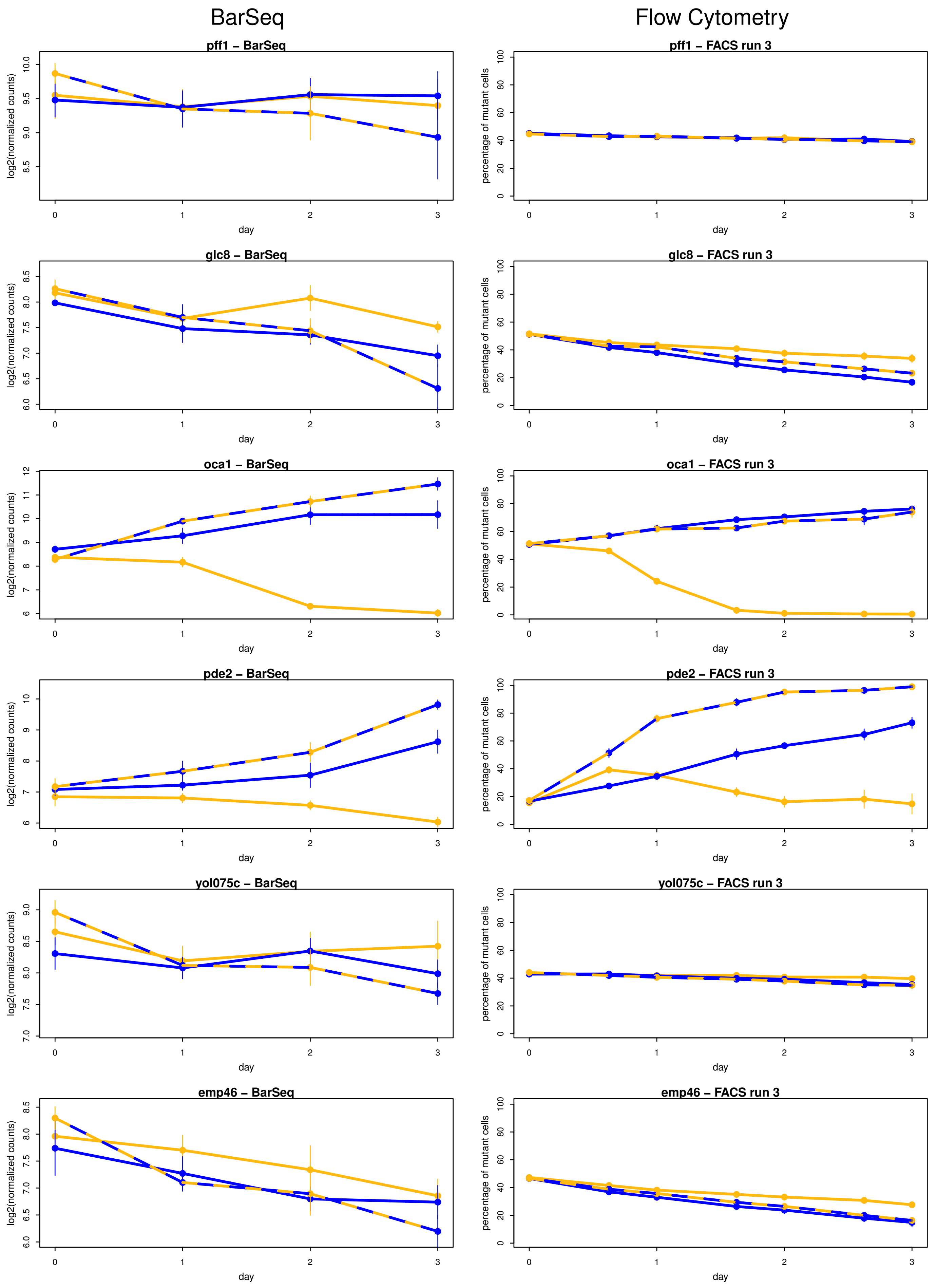
BarSeq
Flow Cytometry
pff1 – BarSeq
pff1 – FACS run 3
glc8 – BarSeq
glc8 – FACS run 3
oca1 – BarSeq
oca1 – FACS run 3
pde2 – BarSeq
pde2 – FACS run 3
yol075c – BarSeq
yol075c – FACS run 3
emp46 – BarSeq
emp46 – FACS run 3
log2(normalized counts)
percentage of mutant cells
day

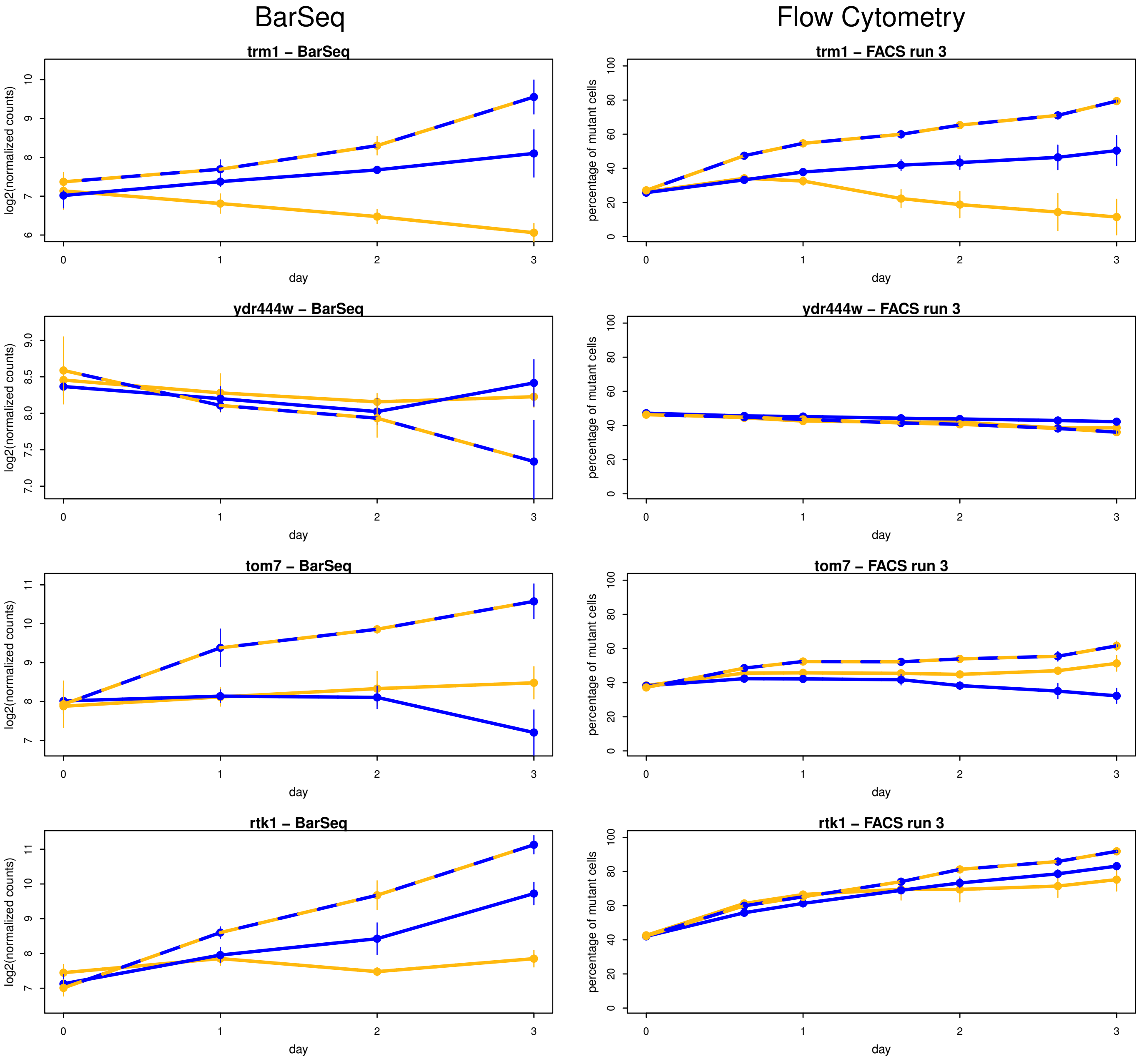
BarSeq
Flow Cytometry
trm1 – BarSeq
trm1 – FACS run 3
ydr444w – BarSeq
ydr444w – FACS run 3
tom7 – BarSeq
tom7 – FACS run 3
rtk1 – BarSeq
rtk1 – FACS run 3
log2(normalized counts)
percentage of mutant cells
day

Appendix Figure S3. Detection of Antagonistic Pleiotropy. Each dot corresponds to one mutant. Coordinates correspond to median fitness values of replicate populations grown in N (*n=3*) or S (*n=4*) condition. Oblique line: y=x. Red, AP mutants.

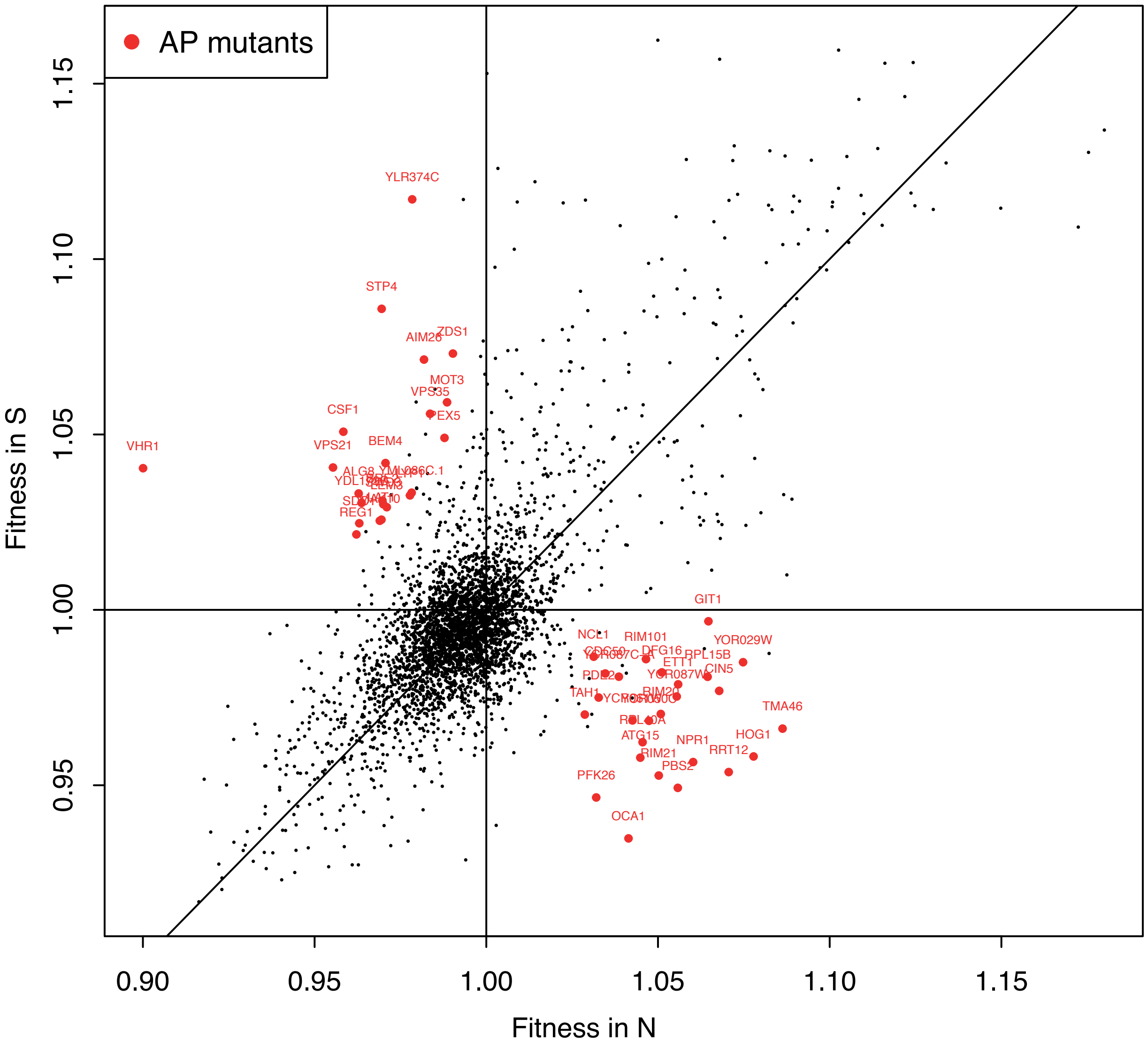
AP mutants
Fitness in S
Fitness in N
0.90
0.95
1.00
1.05
1.10
1.15
YLR374C
STP4
ZDS1
AIM26
MOT3
VPS35
PEX5
CSF1
VHR1
VPS21
BEM4
REG1
GIT1
NCL1
RIM101
YOR029W
DFG16
RPL15B
ETT1
CIN5
YOR087W
PDE2
TAH1
TMA46
ATG15
NPR1
HOG1
RIM21
RRT12
PBS2
PFK26
OCA1

Appendix Figure S4. BAR-seq fitness profile of mutants of the Set1/COMPASS complex. (**A**) For each mutant of the complex available in our data is shown their time-course abundance (left) and their fitted Generalized Linear Model (right), as in Figure 1. (**B**) Schematic representation of the Compass complex (based on (Soares *et al*, 2014)) with colors corresponding to the level of fitness inhomogeneity of each member of the complex.

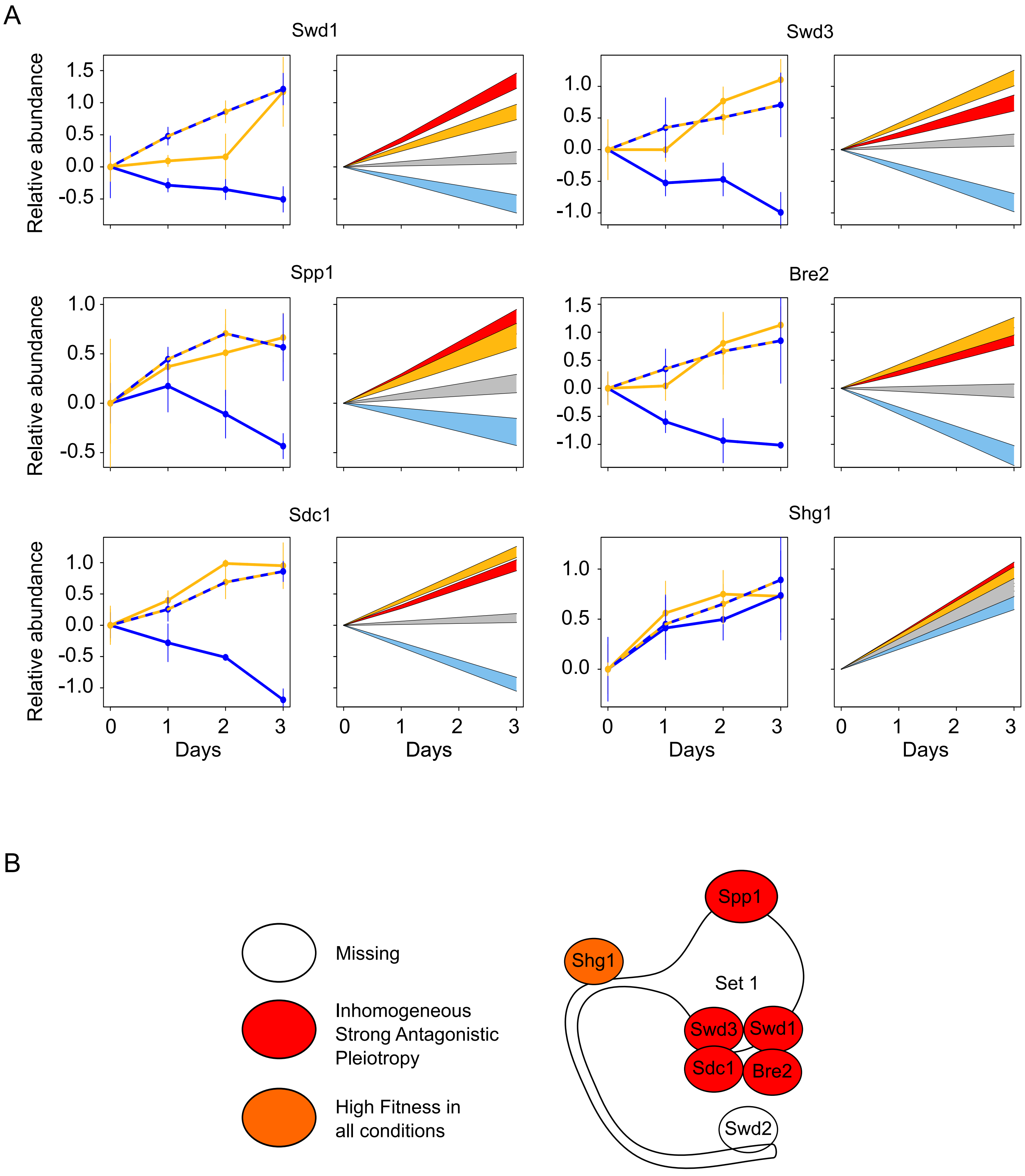

A
Swd1
Swd3
Spp1
Bre2
Sdc1
Shg1
Relative abundance
Days
B
Missing
Inhomogeneous Strong Antagonistic Pleiotropy
High Fitness in all conditions
Spp1
Shg1
Set 1
Swd3
Swd1
Sdc1
Bre2
Swd2

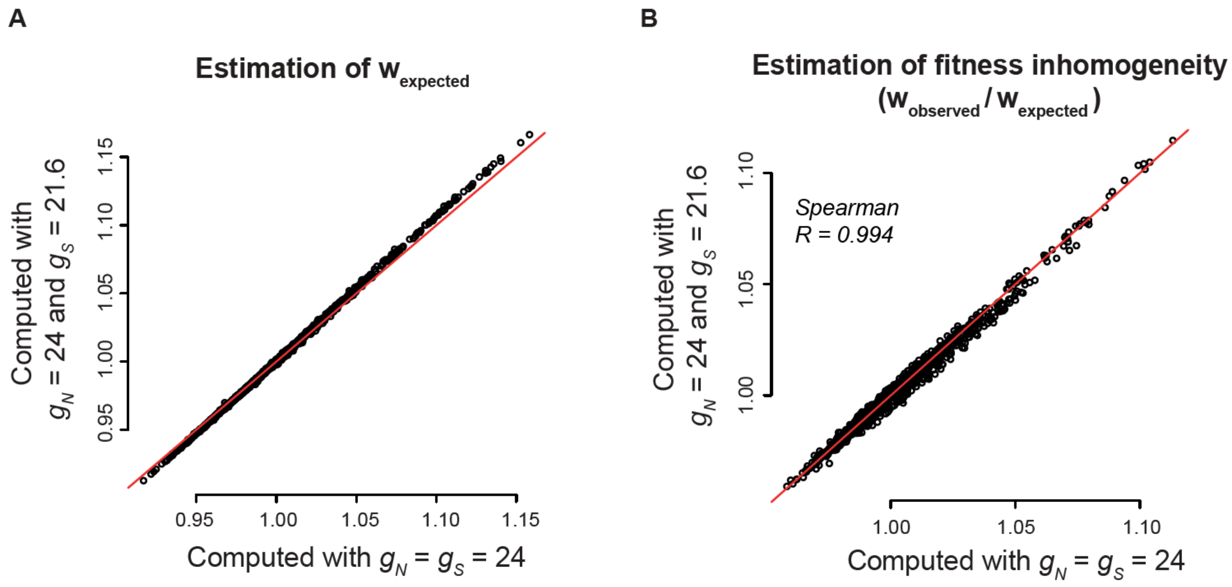

Appendix Figure S5. Effect of correcting for a lower generation time in S. For the salt experiment, fitness values in steady S were recomputed with a number of generations of 21.6 instead of 24. (**A**) Resulting values of fitness in case of homogeneity ($w_{expected}$) as compared to initial values. (**B**) Resulting values of fitness inhomogeneity ($w_{observed}$ / $w_{expected}$) in the 6h-periodic regime of the salt experiment as compared to initial values. Red lines: $y = x$.

Appendix Table S1. Number of deletion mutants having significant fitness inhomogeneity in the 6h-periodic regime, based on Generalized Linear Model.

A. Periodic salt stress

False Discovery Rate	Significant mutants	Deduced number of true positives
0.5	3568	1784
0.4	3438	2063
0.3	2941	2059
0.2	2497	1998
0.1	1934	1741
0.05	1603	1523
0.01	1050	1039
0.001	647	646
1e-4	456	456
1e-5	341	341
1e-6	257	257

B. Periodic methionine availability

False Discovery Rate	Significant mutants	Deduced number of true positives
0.5	3568	1784
0.4	3281	1967
0.3	2799	1960
0.2	2313	1850
0.1	1752	1577
0.05	1387	1318
0.01	800	792
0.001	383	382
1e-4	217	217
1e-5	134	134
1e-6	77	77

Appendix Table S2. Gene Ontology analysis. Enrichment for GO terms were searched using FunSpec (Robinson *et al*, 2002) (last updated in July 2011) at Bonferroni-corrected *P*-value < 0.01.

A. Gene Deletions with significant fitness inhomogeneity: higher fitness under the 6h-periodic regime than expected: *n*=312 input genes.

Functional Category	*P*-value	Enrichment	Genes concerned
Transcriptional control	4.97e-07	44 out of 426	HHF1 HHT1 LRE1 CDC50 SOK1 GIS1 SWR1 LRS4 YHP1 UBP3 CHD1 SWP82 MIG1 YAP3 STP2 RTT107 SKN7 ASG1 TPK1 RSF2 MSN4 SIS2 BRE2 SPT8 HOG1 LEU3 DAT1 MSN2 SUB1 NAT4 GAT2 CRZ1 SPS18 LAP3 YAP7 HAL9 CIN5 ISW2 EGD1 SSN3 SGF11 TPK2 SUT2 MED1
cAMP/cGMP mediated signal transduction	7.7e-06	6 out of 12	SOK1 TPK1 RAS2 RGS2 PDE2 TPK2

B. Gene Deletions with significant fitness inhomogeneity: lower fitness under the 6h-periodic regime than expected: *n*=144 genes.
No enrichment detected.

C. Gene deletions with high fitness inhomogeneity and moderate expected fitness (blue dots in Fig. 3A-C): ***n*=43 genes.**

Functional Category	*P*-value	Enrichment	Genes concerned
osmosensing and response	7.49e-08	6 out of 35	PBS2 SSK1 SSK2 HOG1 HAL9 CIN5

D. Gene Deletions with significant antagonistic pleiotropy: *n*=48 genes.
Enrichments detected.

Functional Category	*P*-value	Enrichment	Genes concerned
COMPASS Complex (Cellzome)	1.85e-05	3 out of 8	SWD3 SDC1 BRE2

E. Gene Deletions with significant transgressivity (extremely high fitness under the 6h-periodic regime): *n*= 55 genes.

Functional Category	*P*-value	Enrichment	Genes concerned
cAMP/cGMP mediated signal transduction	2.03e-06	4 out of 12	TPK1 RAS2 RGS2 PDE2

F. Gene Deletions with significant transgressivity: (extremely low fitness under the 6h-periodic regime): *n*= 23 genes.

No enrichment detected.

Appendix Table S3. Number of Antagonistic Pleiotropic mutants detected at various stringency.

Number of replicates where the mutant is observed AP	Expected by chance only	Actual
0	3568.00	3568
1	563.45	447
2	32.01	101
3	0.54	48

Appendix Table S4. Deletions of genes and pseudogenes used to infer Wild-Type fitness.

Open Reading Frame	Name
YIR044C	YIR044C
YIL170W	HXT12
YIR043C	YIR043C
YCL074W	TY5A
YDL227C	HO
YLL017W	YLL017W
YDR134C	CCW22
YLL016W	SDC25
YIL167W	SDL1
YIL168W	YIL168W

Appendix Table S5. Number of mutants showing transgressive fitness at 6h-period of salt fluctuations.

Number of replicates where the mutant is observed transgressive	Expected by chance only	Actual
0	7136	7136
1	1138.44	872
2	73.30	229
3	2.24	78
4	0.02	35

Appendix Table S6. DNA primers used in this study.

Name	Sequence (5'-3')
1D11	AGGGAAGACAAGCAACGAAACGT
1D12	CCAGCCCATATCCAACTTCCAAT
1O21	TGAATTGTAATACGACTCACTATAGGGCGAATTGGAG CTCCACCGCGGTGGCGGCCGCCAGCTGAAGCTT
1O22	GATATCGAATTCCTGCAGCCCGGGGGATCCACTAGTTC TAGAGCGGCCGCGCGGCCGCATAGGCCACTGT
1O27	AAATTAGAGCTTCAATTTAATTATATCAGTTATTACCCGGGGTTAATTAA GCGCCAAAGGTGCAGAATTCAT
1O28	ACAAAACATTCTGTGAAGTTGTTCCCCCAGGAATTCGAGCTCGTTTAAAC CAAGACCCGTGGTCCATTCCATTT

Appendix Table S7. Yeast strains used in this study.

Name	Genotype	Source
BY4716	*MATalpha lys2Δ0*	(Brachmann *et al*, 1998)
BY4742	*MATalpha his3Δ1 leu2Δ0 lys2Δ0 ura3Δ0*	(Brachmann *et al*, 1998)
BY4743	*MATa/MATalpha his3Δ1/his3Δ1 leu2Δ0/leu2Δ0 lys2Δ0/LYS2 met15Δ0/MET15 ura3Δ0/ura3Δ0*	Euroscarf (Y20000)
GY1735	*MATa/MATalpha his3Δ1/his3Δ1 leu2Δ0/leu2Δ0 lys2Δ0/LYS2 met15Δ0/MET15 ura3Δ0/ura3Δ0 hoΔ::kanMX4/HO*	This study
GY1738	*MATa/MATalpha his3Δ1/his3Δ1 leu2Δ0/leu2Δ0 lys2Δ0/LYS2 met15Δ0/MET15 ura3Δ0/ura3Δ0 hoΔ::kanMX4-GFP/HO*	This study
GY1804	*MATa/MATalpha his3Δ1/his3Δ1 leu2Δ0/leu2Δ0 lys2Δ0/LYS2 met15Δ0/MET15 ura3Δ0/ura3Δ0 tom7Δ::kanMX4/tom7Δ::kanMX4*	Euroscarf (Y37217)
GY1821	*MATa/MATalpha his3Δ1/his3Δ1 leu2Δ0/leu2Δ0 lys2Δ0/LYS2 met15Δ0/MET15 ura3Δ0/ura3Δ0 YOR360C::kanMX4/YOR360C::kanMX4*	Euroscarf (Y31657)
GY1921	*MATa/MATalpha his3Δ1/his3Δ1 leu2Δ0/leu2Δ0 lys2Δ0/LYS2 met15Δ0/MET15 ura3Δ0/ura3Δ0 tom7Δ::kanMX4/tom7Δ::kanMX4 HO/hoΔ::URA3-TOM7*	This study
GY1929	*MATa/MATalpha his3Δ1/his3Δ1 leu2Δ0/leu2Δ0 lys2Δ0/LYS2 met15Δ0/MET15 ura3Δ0/ura3Δ0 hoΔ::URA3-PDE2/HO pde2Δ::kanMX4/pde2Δ::kanMX4*	This study
GY1958	*MATa/MATalpha his3Δ1/his3Δ1 leu2Δ0/leu2Δ0 lys2Δ0/LYS2 met15Δ0/MET15 ura3Δ0/URA3 hoΔ::kanMX4/HO*	This study
GY1961	*MATa/MATalpha his3Δ1/his3Δ1 leu2Δ0/leu2Δ0 lys2Δ0/LYS2 met15Δ0/MET15 ura3Δ0/URA3 hoΔ::kanMX4-GFP/HO*	This study

REFERENCES

Brachmann CB, Davies A, Cost GJ, Caputo E, Li J, Hieter P & Boeke JD (1998) Designer deletion strains derived from Saccharomyces cerevisiae S288C: a useful set of strains and plasmids for PCR-mediated gene disruption and other applications. *Yeast* **14:** 115–32

Robinson MD, Grigull J, Mohammad N & Hughes TR (2002) FunSpec: a web-based cluster interpreter for yeast. *BMC Bioinformatics* **3:** 35

Soares LM, Radman-Livaja M, Lin SG, Rando OJ & Buratowski S (2014) Feedback Control of Set1 Protein Levels Is Important for Proper H3K4 Methylation Patterns. *Cell Rep.* **6:** 961–972
